# Socio-epidemiological determinants of 2002 plague outbreak in Himachal Pradesh, India: a qualitative study

**DOI:** 10.1186/1471-2458-14-325

**Published:** 2014-04-08

**Authors:** Sonu Goel, Harvinder Kaur, Anil Kumar Gupta, Umesh Chauhan, Amarjeet Singh

**Affiliations:** 1 Health Management, School of Public Health, PGIMER, Chandigarh, India; 2 School of Public Health, PGIMER, Chandigarh, India; 3 Department of Hospital Administration, PGIMER, Chandigarh, India

**Keywords:** Gujjars, Himachal Pradesh, Hunting, Lifestyle, Plague, Ethno-aetiology and social epidemiology

## Abstract

**Background:**

This qualitative investigation was conducted to determine the socio-epidemiological factors related to the plague outbreak (2002) in Himachal Pradesh (HP), India.

**Methods:**

The data for socio-epidemiological factors related to the plague outbreak (2002) in HP was obtained from residents through 150 in-depth Interviews (IDI) and 30 Focus Group Discussions (FGD) during six visits (from May 2011 to April 2012) by the research team. Natives, health officials and the nomadic population were interviewed. According to their opinion and viewpoints data was collected and their lifestyle and hunting practices were studied in detail. Tape recorders were used during various FGDs and IDIs. The interviews and FGDs were later transcribed and coded. In-depth analysis of the recorded data was done using an inductive thematic analysis approach.

**Results:**

The study reports that the outbreak in 2002 in a few villages of Himachal Pradesh was that of plague and it occurred by the contact of an index case with wild animals after hunting and de-skinning. The first wave of plague transmission which took 16 lives of residents was followed by a second wave of transmission in a ward of a tertiary care hospital where one visitor acquired it from relatives of the index case and succumbed. The life-style practices of residents (hunting behavior, long stay in caves and jungles, overcrowding in houses, poor hygiene and sanitation, belief in ‘God’ and faith healers for cure of diseases) was optimal for the occurrence and rapid spread of such a communicable disease. The man-rodent contact is intensified due to the practice of hunting in such a rodent-ridden environment. The residents harbor a strong belief that plague occurs due to the wrath of gods. Various un-reported outbreaks of plague were also observed by officials, residents and old folk. The persistence of plague in HP is favoured by its hilly terrain, inaccessible areas, inclement weather (snow) in winters, unhygienic lifestyle, hunting practices of residents, and treatment practices through faith healers.

**Conclusions:**

This study suggests that the lifestyle of the natives of HP and other socio-epidemiological factors played a role in the outbreak of plague in that area.

## Background

Plague continues to remain endemic [1] even in this day and age across various parts of the world [2] including India [3]. Although plague is an internationally notifiable disease, it has ceased to exist in the list of priority diseases circulated by the Government of India and other agencies. In India, plague outbreaks were reported in the year 1994 in the two states of Beed (Maharashtra) and Surat (Gujarat). A local outbreak of plague had occurred in Himachal Pradesh (2002) and Uttaranchal (2004). Earlier, it was maintained that plague had ceased to exist in India after 1966 [3]. Despite repeated outbreaks, the disease is not given much importance in medical undergraduate and postgraduate teaching. Most practicing doctors are not aware of the manifestation and management of plague.

The extensive search of literature revealed that most of the research on plague focuses largely on the proximate causes (i.e. microbial pathogens), at the expense of the wide socio-epidemiological contributors. Very few authors have discussed the social aspects (other than the impact of the disease on the economy) of the disease. Kreiger (2001) [4] has written that a study of biological and social determinants is required for an in depth understanding of the recurrent outbreaks of plague in India. The importance of social factors as causes of disease has been well established. Indeed, questions of social causation of disease have been asked throughout the history of public health. The present study was therefore undertaken to ascertain various socio-epidemiological factors related to the 2002 outbreak of plague in Himachal Pradesh that occurred almost a decade later than earlier outbreaks of similar disease in India.

## Material and methods

This qualitative study was conducted in the area of Himachal Pradesh (HP) that was affected during the 2002 plague outbreak. Data on socio-epidemiological factors related to the 2002 plague outbreak in HP was obtained from the residents of the Hatkoti-Jubbal and Nerua-Chaupal belt of District Shimla on an interview Performa during six visits (from May 2011 to April 2012) by the research team in these regions. These people were selected in order to gather maximum information about the knowledge and opinion of a broad base of stakeholders on the etiology, proneness, sustenance, spread, control and prevention of plague and the on-guard measures adopted by them against plague.

A total of 30 FGD and 150 IDI were conducted amongst affected families, natives, Gujjars (nomadic population) and health officials (doctors, pharmacists, lab technicians, nurses and ANMs) involved during the 2002 (HP) plague outbreak. Each FGD lasted for around 4 hours and one IDI for one and half hour. A tape recorder was used as required during various FGDs and IDIs. Field notes were taken manually on diaries for qualitative data analysis. The hunting behavior of villagers and the extent of non-vegetarian food consumption pattern of the local people was also noted especially on snowfall days when getting regular food is difficult. A lifestyle profile of the natives of the affected villages was created. Their opinion on the repeated occurrence of plague in their area, the role of other factors especially rats in the spread, type and pattern of plague, local terminology for the disease, treatment seeking behavior, changes in health seeking behavior after the 2002 outbreak etc. were also catalogued. Further, attempts were made to understand the myths and traditional healing practices including ideas of ethno-aetiology and periodicity of various infections and diseases of Himachal Pradesh from the local residents. For each category, the interviews continued until ‘saturation’ occurred i.e. no further information can be elicited about the category. The interviewees were free to talk about the issues in their mother tongue. The IDI and FGD were later transcribed and segmented into paragraphs. Each paragraph was examined in-depth to generate ‘categories’ and merge them into larger categories. At the end of the interview the categories were shared with the participants to ascertain if they were a true reflection of their opinion. The coding reliability was thoroughly checked by independently asking two trained investigators to slot them in core themes, which generated a high (92%) correspondence. The data was analysed using an inductive thematic analysis approach. Apart from this, available literature and reports on plague (after the 1994 Surat outbreak) were also reviewed.

Written informed consent was taken from the respondents and the study protocol was duly approved by the Ethics Committee of the Postgraduate Institute of Medical Education & Research (PGIMER), Chandigarh, India. We also obtained permission from the Director General of Health Services and Wild Life Authority of the state of Himachal Pradesh to conduct the study.

## Results

The results are broadly summarized into four broad themes generated during the study: information about 2002 outbreak, lifestyle of natives of Himachal Pradesh (HP), lifestyle of Gujjars, and management of 2002 outbreak.

### The 2002 plague outbreak

**Figure 1:**
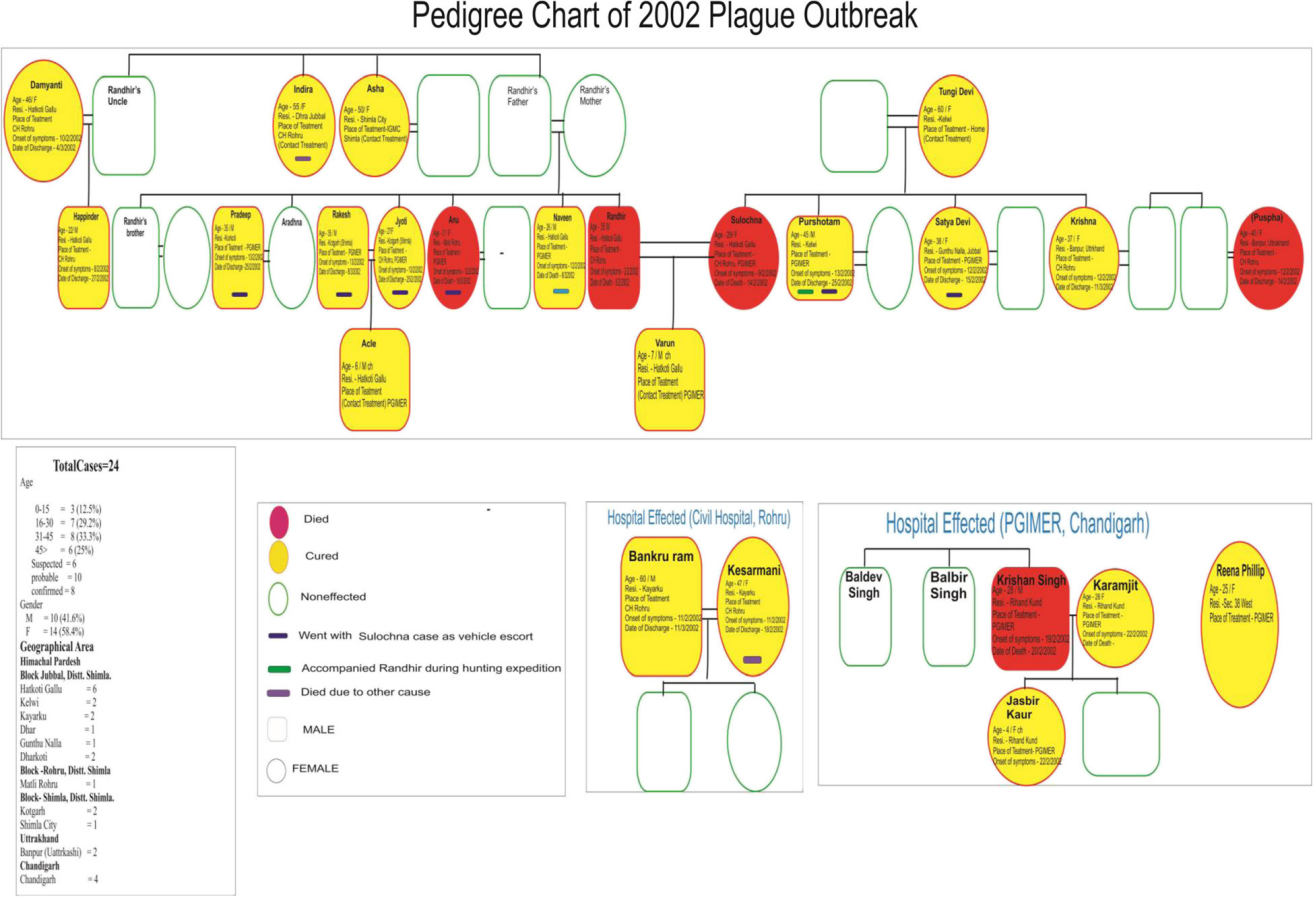
Pedigree Chart of 2002 plague outbreak in Himachal Pradesh, India.

The plague outbreak in 2002 started when the index case Randhir (35-year-old male) residing in Hatkoti village of Shimla District in HP visited his mother-in-law’s place at Kelwi village on the eve of ‘Bakar Eid’ (local festival) in 2002 with his wife Sulochana. There was unprecedented heavy snowfall (54 cm snow on February 2, 2002) during that period. Randhir had gone for hunting along with his brother-in-law Purshottam in a nearby forest called Mural on January 25, 2002 at a height of approximately 500–600 m from his house. They hunted a wild bird (probably “Munnal or Jangli Murga”) skinned it and consumed its meat. However, it came out during various FGD’s that contrary to the popular belief that they had hunted a wild bird, Randhir and Purshotam had found a sick wild cub in the forest which they had brought to their home and only Randhir had done its skinning, from where probably he had acquired the infection. On February 2, 2002 Randhir developed high grade fever with chills and rigors. Gradually he developed chest pain and the illness continued to progress with the onset of hemoptysis. His family members took him to the Civil Hospital at Jubbal where he was diagnosed as suffering from pneumonia and was given some injections and oral medication. As there were no signs of recovery he was taken to the Civil Hospital at Rohru on February 4, 2002 at 10:10 pm. He was suffering from fever and pain and was frequently vomiting blood. He developed haematuria and gradually progressed into a state of shock and died on 5 February, 2002. Till death, he was kept in the general ward of the Civil Hospital (Rohru) along with other patients. Plague was not suspected even after his death. The pedigree chart of plague affected victims of 2002 plague outbreak in Himachal Pradesh is shown in Figure 1’).

Three days after Randhir’s death (Feb 8, 2002), Sulochana (Randhir’s wife) also developed similar symptoms. She was taken to the Civil Hospital, Rohru. There, Dr. Ramlal, the Civil Surgeon of Rohru suspected that she was suffering from plague and referred her to a premier tertiary care institute of North-Western India (PGI, Chandigarh). She was escorted by Naveen, Asha Devi, Purshottam, Anu, Rakesh, Pardeep, Satya Devi and Jyoti. Sulochana died at PGI Chandigarh. Doctors at PGI could not diagnose plague. After three days, Satya Devi (Sulochna’s sister) and Anu (Randhir’s sister) developed similar symptoms. Anu died after five days. In a time span of one week all the people who had escorted Sulochana developed these symptoms.

Later, several family members and relatives, who had come in contact with the index case and/or his wife during their illness at home, hospital or during cremation activities, were admitted to PGI, Chandigarh between 9 and 13 February 2002. A total of sixteen people were reportedly diagnosed as cases of pneumonic plague and succumbed to the illness in the first wave of plague transmission. The disease was confined to three houses in a hamlet in “Hatkoti” village and all the deceased were relatives of the index case. On 13 February 2002, the outbreak was diagnosed as a ‘highly communicable disease’ (secondary attack rate of 75%) with a short incubation period (1–4 days) and high case fatality spreading through droplet transmission [5]. After the bacteriological confirmation of the diagnosis on 16 February 2002, it was declared as a plague outbreak on 20 February 2002.

The second wave of transmission occurred when a 28 year old male visitor of a patient admitted to the Emergency Room of PGI, Chandigarh reportedly came in contact with the relatives of the index case on 10 February 2002. On 17 February 2002, he developed fever with chills and chest pain. Next day, he developed haemoptysis along with episodes of vomiting. He sought treatment from a government hospital, as well as a private clinic in Chandigarh. On 19 February 2002, he was brought to the Emergency Room of PGI in a critical condition where he died in the early morning of 20 February 2002. During the course of his illness and cremation he transmitted the disease to his wife Karamjit Kaur, daughter Jasbir Kaur and the nurse Reena Philip attending to him at the private clinic.

### Lifestyle of natives of Himachal Pradesh

Most of the natives in Himachal Pradesh knew about the 2002 plague outbreak. Locally, it is called ‘Byadh’. They believe that plague is spread by rats (Moosh and Ghuns in the local language), through the bite of flea (pissu) living on a rat’s body and also by consumption of infected meat.

The natives of HP earn their livelihood mostly from agriculture, mainly the apple orchards. Some work as daily wagers. During summers mostly pulses and vegetables are consumed. People also consume non vegetarian food during winters. They also eat ‘purana’ meat (Old meat). It is believed that the older the meat, the tastier it is. Meat is stored for a long period by hanging the skin intact with nails on a hook suspended from the roof of the room. Proper hygiene is not observed and people remain without bathing for 20–25 days. People don’t bother about immunization. They have strong faith in their own ‘Devi-Devta’ (gods). Each house and every village worships its own particular god. All local people are highly superstitious. They visit ‘Tantriks’ (faith healers) for curing diseases.

The agricultural area of villages in Himachal Pradesh is attached to dense forests. Semi-domestic rats are be found inside houses and wild rodents are present in high density in the forest. Local people believe that the environment plays a major role in the spread of plague. According to them, the months of February and March are the most favorable time period because rats come out of their burrows in search of food. Some villagers believe that plague outbreak occurs due to the wrath of God and other supernatural forces.

**Figure 2:**
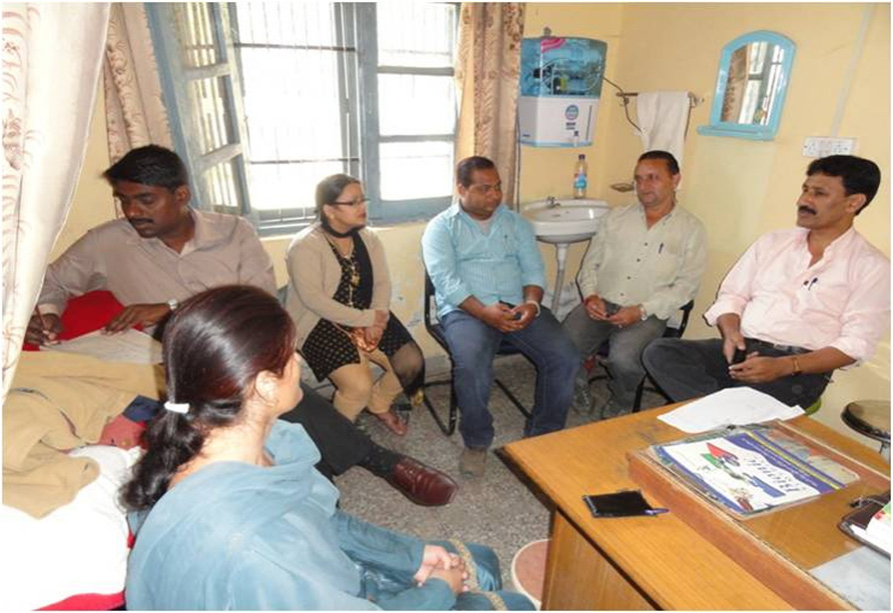
Interaction with health officials involved during 2002 Plague outbreak in Himachal Pradesh, India.

From the present study, the fact emerged that hunting is mostly done during winters in the dense forests e.g. Mural Forest. Hunting is called ‘Adecka’. Some of the residents have guns and a few have specially trained dogs for hunting purposes. Very few people have licensed guns and the rest use unlicensed guns or other tools for killing purposes even though all these are strictly banned by the government. People go in groups of 10–12 and stay there in ‘udaar’ (caves) or tents. This is their way of relaxation. They seemed to hide the details on this issue from investigators due to the so-called ‘ban on hunting’. Though banned, hunting by the men folk is likely to continue in a scattered manner in HP. Staying in the hills for days together increases their exposure to rats/burrows (in caves) (Figure 2).

### Management of various plague outbreaks in HP

**Figure 3:**
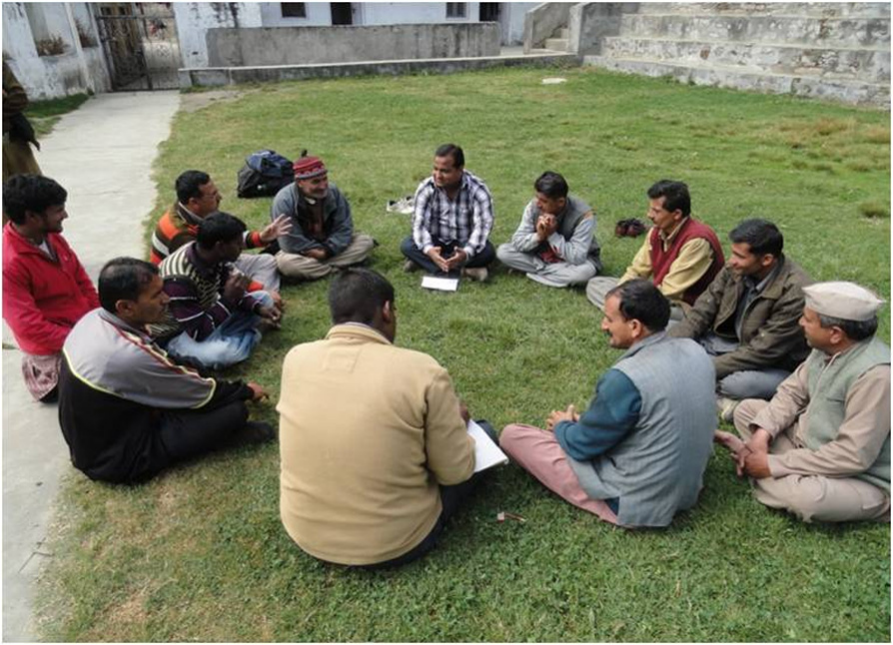
Focus Group Discussion with affected families and natives of villages affected during 2002 plague outbreak.

The former Programme Director of the World Health Organization (WHO) informed the investigating team about a Plague outbreak in Himachal Pradesh that occurred in the year 1948 in which around 200 deaths occurred. Some doctors who were in service in the 1980s told the investigators about undocumented plague outbreaks (1983 in Tangnu and Jaangli villages of Tehsil Chiragaon, 1984 in Mandol village, 1985 in Rohru). According to a few respondents, another suspected plague outbreak has been reported in 1971–72 and in Samarkot, as recently as September 1992. One of the retired doctors told the investigating team that people in these areas ate roasted rats which could be the reason for the plague epidemic. People believe that rat meat cures chest infection and is beneficial during winters. Dr. Retola, who was the Chief Medical Officer of the Rohru Civil Hospital (when plague occurred in 1983), had warned the authorities about the possibility of its recurrence through an official letter in 1996. He said that plague has a cyclic recurrence every 10–15 years. The natives of the area also reported that the team from Delhi didn’t visit the interior/remote areas (due to intense cold) during the 2002 outbreak. They just took some samples from semi-domestic rodents and returned to Delhi. The government report states that the plague surveillance units set up in Himachal Pradesh after the 2002 outbreak were not functioning efficiently when its evaluation was done in 2011 (Figure 3).

### Lifestyle of Gujjars

**Figure 4:**
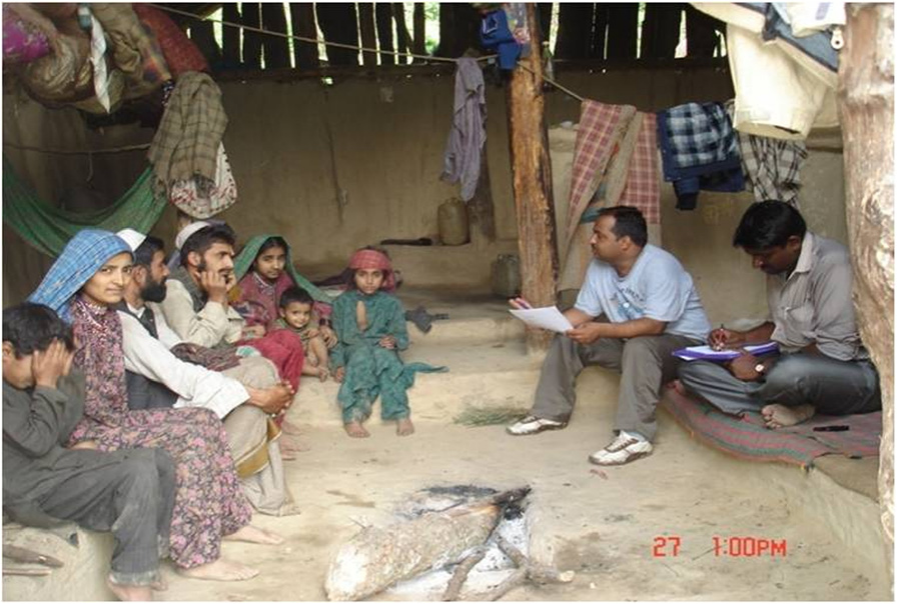
Life style of Gujjars (nomadic population) in the study area.

Gujjars are Muslim migrant shepherds who visit the HP forest areas every year. They start migrating from Uttarakhand in April and reach Himachal in May every year. They again start migrating from Himachal in the last week of September and reach Uttarakhand in October. The Gujjars were aware of the outbreak which occurred in 2002 in Himachal Pradesh. However, they were uncertain about the disease. They are aware that during plague, the body temperature shoots up. They call it a disease of cold (Thande ki Bimari). They believe that the plague outbreak starts when a person visits the forest for hunting where he consumes the meat of a bird and stays at a hunter’s place.They have seen some red and some white colored rats. They know the sites of rat burrows. On request, they helped the team in catching them from their burrows. Wild rats are called “Punjali Chuhey” or “teer ki thand ka chuha” (the rat found in intense cold weather). Most of the Gujjars, follow a primitive lifestyle and live in “kutcha” hutments (thatched). They abandon their hutments also known as “deras” when they leave for Uttarakhand (Figure 4).

## Discussion

The study revealed that the outbreak in 2002 in a few villages of Himachal Pradesh was that of plague which occurred due to the contact of the index case with wild animals after hunting and de-skinning. However, it still remains unclear whether it occurred due to de-skinning or consumption of meat of wild animal. The first wave of plague transmission which took 16 lives of residents was followed by a second wave of transmission in a ward of a tertiary care hospital where one visitor acquired it from the relatives of the index case and succumbed to it. The life-style practices of residents (hunting behavior, long stay in caves and jungles, overcrowding in houses, poor hygiene and sanitation, belief in ‘God’ and faith healers for cure of diseases) was well suited for the occurrence and rapid spread of communicable diseases. The hunting time period and rodent activity makes the residents even more prone to man-rodent contact. There is a strong belief among residents that occurrence of plague is due to the wrath of the gods. Various un-reported outbreaks of plague were also recorded by high level officials, which were in consonance with the views of residents and old folk.

Globally, the number of plague cases reported by the WHO decreased to an all-time low of 200 in 1981 [6]. The last laboratory confirmed human case in India was reported in 1966 from Karnataka [7]. A global trend of decreasing plague incidence from 1950 to 1980 was followed by decreased financial support for appropriate diagnostic facilities, lowered interest of the health care authorities and ultimately the deterioration of surveillance systems for the disease. So much so that many countries were no longer capable of making a laboratory diagnosis of plague in the 1990s. For example, in 1994 when an outbreak of plague occurred in Western India [8], lack of laboratory capacity for diagnosis led to confusion as to the cause of the outbreak and panic within the population.

Endemic foci in the Himalayas have been there since long. Plague infection was known to exist in the Kumaon and Garhwal districts even in 1823. Plague outbreaks have also occurred at short intervals in HP during the late 1950s and early 1960s [9]. The persistence of plague in Himachal Pradesh is favored by its hilly terrain, inaccessible area, inclement weather (snow) in winter. Local traditions (traditional healing, funeral ceremonies), and the social conditions of the population (general resistance of individuals, ignorance of hygiene measures, type and structure of houses) combined with low access to medical care further aggravate the situation.

In Surat (Gujarat), a combination of heavy rains and clogged sewers led to unhygienic conditions and a number of decaying animal carcasses precipitated the plague epidemic by providing a breeding ground for rats and fleas [10]. Similar re-emergence of plague in Madagascar was due to the dramatic reduction of the country’s resources devoted to public hygiene and the low socio-economic conditions of the majority of the population [11]. So, it is clear that socio-environmental factors and poor financial allocation were mainly responsible for creating conditions conducive for the occurrence of plague.

The environmental factors again provided favorable conditions for the occurrence of plague in Beed (Maharashtra) in 1994. The storage of grain in damaged homes that people had abandoned after the 1993 earthquake in Maharashtra was an attractive food supply for rats. Further, limited health care for the poor and an insufficiently funded public health system prevented rapid identification and treatment of outbreaks. Finally, the religious practice of rat worship increased the potential for exposure to humans from infected rat fleas [2]. Thus; social factors were responsible for the spread of plague there.

Another social determinant of vector borne disease is human encroachment upon previously uninhabited environments, virgin forests and jungles. This keeps supplying man with new virulent diseases in company with exotic plants and animals. Humans are at risk of acquiring plague whenever they visit infected areas as hunters, trappers or nomads. Though banned, hunting is likely to continue in a scattered manner in HP. Staying in the hills for days together increases their exposure to rats/burrows (in caves). It has been reported by experts that people can be infected directly from a plague infected rodent or other animal while handling, skinning or cutting up the meat [5]. The similar situation of de-skinning of the wild animal in the present study could have led to infection from plague.

The lifestyle conditions of Gujjars were also explored in the present study. They live deep in forests away from the mainland natives. They could therefore be a potential link between any source of infection in forests and the HP natives. They have also been said to regularly visit the caves in deep forests. Therefore, they are regularly exposed to the microclimate of rats/cave etc. This way, their community can be a ‘sitting duck’ for any potential spread of any vaccine preventable disease. Even otherwise too, the Gujjar community is barely covered by the government health services. This coupled with their illiteracy and poor socioeconomic status makes the Gujjar population a potential future source of infection.

Not just the social etiology, plague had a major social impact too. With the possibility that these severe plague cases could occur in neighboring cities or possibly even other countries, tremendous fear rapidly spread beyond Surat and Beed. In response to this threat, several countries closed their borders to travelers and cargo from India and stopped all air flights to and from India [12,13]. The WHO estimated that the outbreak cost India some $2 billion. The lack of a definitive diagnosis in this outbreak also highlighted the plight of medical education in India, as how seriously bacteriology was neglected in the Indian medical, health and scientific community [14].

Himachal Pradesh is known for its widespread apple orchards and high quality apple produce. Apples from Himachal are transported to the other parts of the country. The apple industry is worth Rs.1, 500 crore (around $330 million). There is a possibility that the plague bacilli may spread due to trade and transport of apples from Himachal Pradesh to other parts of the country via rodents which may inhabit trucks that are used during apple transportation. This aspect was highlighted in the analysis of the epidemic wave of plague in Mahajanga and Madagascar (1991-1992) [11]. This aspect needs to be investigated further for a possible link between the plague outbreak in Maharashtra, Gujarat and HP.

The delay in the initiation of effective treatment of plague cases during the 2002 HP outbreak was a major factor that led to the spread of the disease [16,17]. This can be linked to the prevailing global focus on evidence-based medicine in the health care set up. In the 2002 outbreak too, confirmed diagnosis was delayed by eight days. The insistence by health providers on lab diagnosis before formally declaring it as an ‘outbreak’ weakened the management of the episode. As a result, public health response was delayed leading to the spread of the disease. In diseases of a sensitive nature like plague remedial measures should have been initiated the moment it was suspected.

Plague epidemiology should be given due emphasis in medical undergraduate teaching. Surveillance needs to be intensified when there are other ecological changes such as earthquake, flood and heavy rainfall and especially if human habitation is in close proximity to the burrows of wild rodents, as the risk of transmission of infection to human beings increases in such situations [3]. It is also suggested that vulnerable areas should be identified. In most of the endemic countries, plague occurs in inaccessible areas where qualified personnel, material for collection of samples or laboratory means are scarce [11]. The health authorities of the area should be regularly on guard.

## Conclusion

Given the complexity of its life cycle, including the number and variety of potential animal and vector hosts involved, it is unlikely that plague will ever be completely eradicated by human endeavor [16]. It needs to be realized that plague is thoroughly entrenched in widespread zoonotic loci that are unlikely to be eliminated, at least in hilly areas. The recent outbreaks in India are a reminder that its resurgence merely requires favorable environmental and public health conditions.

The strength of the study is the large sample size involving wide stakeholders. The ‘saturation’ concept of qualitative study was used. However, the limitations were lack of corroboration of findings with quantitative data and inability to explore a few contentious findings where consensus couldn’t be reached. However, despite the limitations, the study has identified the role of socio-environmental factors in the occurrence of plague in Himachal Pradesh. In fact, it is the first study which explored the linkage between lifestyle and practices of residents with recurrent plague outbreaks in Himachal Pradesh.

## Competing interests

The authors declare that they have no competing interests.

## Authors’ contribution

Dr. SG Concept, design, manuscript editing, manuscript review, read and approved the final version. Dr. HK Literature search, data acquisition, manuscript preparation, read and approved the final version. Dr. AKG Manuscript editing, manuscript review, read and approved the final version. UC Data acquisition, literature search, read and approved the final version. Prof. AS Concept, design, manuscript editing, manuscript review, read and approved the final version. All authors read and approved the final manuscript.

## Pre-publication history

The pre-publication history for this paper can be accessed here:

http://www.biomedcentral.com/1471-2458/14/325/prepub

## References

[B1] Boisier P Rahalison L Rasolomaharo M Ratsitorahina M Mahafaly M Razafimahefa M Duplantier JM Ratsifasoamanana L Chanteau S Epidemiologic features of four successive annual outbreaks of bubonic plague in Mahajanga, Madagascar Emerg Infect Dis 2002 8 3 311 316 10.3201/eid0803.010250 11927030 PMC2732468

[B2] Clem A Galwankar S Plague: a decade since the 1994 outbreaks in India J Assoc Physicians India 2005 53 457 464 16124356

[B3] Agarwal SP Plague Control in India. Directorate General Health Services 2005 New Delhi, India: Ministry of Health and Family Welfare

[B4] Krieger N A glossary for social epidemiology J Epidemiol Community Health 2001 55 10 693 700 10.1136/jech.55.10.693 11553651 PMC1731785

[B5] Joshi K Thakur JS Kumar R Singh AJ Ray P Jain S Varma S Epidemiological features of pneumonic plague outbreak in Himachal Pradesh, India Trans R Soc Trop Med Hyg 2009 103 5 455 460 10.1016/j.trstmh.2008.11.026 19211117

[B6] Dennis DT Scheld WM, Craig WA, Hughes JM Plague as an emerging disease Emerging Infections 1998 2 Washington DC: ASM Press 169 183

[B7] Gupte MD Ramachandran V Mutatkar RK Epidemiological profile of India: historical and contemporary perspectives J Biosci 2001 26 437 464 10.1007/BF02704746 11779959

[B8] Ramalingaswami V The plague outbreaks of India, 1994. A prologue. Curr Sci 1996 71 781 782

[B9] Sehgal S Bhatia R History of Plague. National Institute of Communicable Diseases 1991 New Delhi, India: Directorate General Health Services

[B10] "Surat: A Victim of Its Open Sewers". New York Times 1994 http://www.nytimes.com/1994/09/25/world/surat-a-victim-of-its-open-sewers.html

[B11] Chanteau S Ratsitorahina M Rahalison L Rasoamanana B Chan F Boisier P Rabeson D Roux J Current epidemiology of human plague in Madagascar Microbes Infect 2000 2 1 25 31 10.1016/S1286-4579(00)00289-6 10717537

[B12] Fritz CL Dennis DT Tipple MA Campbell GL McCance CR Gubler DJ Surveillance for pneumonic plague in the United States During an international emergency: A model for control of imported emerging diseases Emerg Infect Dis 1996 2 30 36 10.3201/eid0201.960103 8964057 PMC2639812

[B13] Burns JF With Old Skills and New, India Battles the Plague. New York Times 1994 http://www.nytimes.com/1994/09/29/world/with-old-skills-and-new-india-battles-the-plague.html

[B14] Mavalankar DV Indian 'Plague' Epidemic: Unanswered Questions and Key Lessons J R Soc Med 1995 88 547 551 8537942 PMC1295353

[B15] Perry RD Fetherston JD Yersinia pestis–etiologic agent of plague Clin Microbiol Rev 1997 10 1 35 66 8993858 10.1128/cmr.10.1.35 PMC172914

[B16] Goel S Singh A Will Plague Continue to Haunt Hilly States of India? The Internet Journal of Health 2007 8 1 avalaible at http://ispub.com/IJH/8/1/10163 (accessed on 11/1/14)

[B17] Kaur H Goel S Sharma Y Kessar RR Singh A Socioenvironmental Etiology of Plague Outbreak in Himachal Pradesh- a Reterospective Enquiry. J Post Grad Med Edu Res 2013 47 2 112 116

